# Severity of coronary artery disease is associated with diminished circANRIL expression: A possible blood based transcriptional biomarker in East Africa

**DOI:** 10.1111/jcmm.18093

**Published:** 2023-12-27

**Authors:** Gokce Akan, Evarist Nyawawa, Bashir Nyangasa, Mehmet Kerem Turkcan, Erasto Mbugi, Mohammed Janabi, Fatmahan Atalar

**Affiliations:** ^1^ Biochemistry Department, MUHAS Genetics Laboratory, School of Medicine Muhimbili University of Health and Allied Sciences Dar es Salaam Tanzania; ^2^ Near East University DESAM Research Institute Mersin North Cyprus Turkey; ^3^ Jakaya Kikwete Cardiac Institute Dar es Salaam Tanzania; ^4^ Department of Electrical Engineering Columbia University New York New York USA; ^5^ Department of Rare Diseases Istanbul University, Child Health Institute Istanbul Turkey

**Keywords:** 9p21.3, ANRIL, circANRIL, coronary artery disease, hsa_circ_0008574, peripheral mononuclear blood cells, polymorphism, transcriptional biomarker, visceral adipose tissue

## Abstract

Antisense Noncoding RNA in the INK4 Locus (ANRIL) is the prime candidate gene at Chr9p21, the well‐defined genetic risk locus associated with coronary artery disease (CAD). ANRIL and its transcript variants were investigated for the susceptibility to CAD in adipose tissues (AT) and peripheral blood mononuclear cells (PBMCs) of the study group and the impact of 9p21.3 locus mutations was further analysed. Expressions of ANRIL, circANRIL (hsa_circ_0008574), NR003529, EU741058 and DQ485454 were detected in epicardial AT (EAT) mediastinal AT (MAT), subcutaneous AT (SAT) and PBMCs of CAD patients undergoing coronary artery bypass grafting and non‐CAD patients undergoing heart valve surgery. ANRIL expression was significantly upregulated, while the expression of circANRIL was significantly downregulated in CAD patients. Decreased circANRIL levels were significantly associated with the severity of CAD and correlated with aggressive clinical characteristics. rs10757278 and rs10811656 were significantly associated with ANRIL and circANRIL expressions in AT and PBMCs. The ROC‐curve analysis suggested that circANRIL has high diagnostic accuracy (AUC: 0.9808, cut‐off: 0.33, sensitivity: 1.0, specificity: 0.88). circANRIL has high diagnostic accuracy (AUC: 0.9808, cut‐off: 0.33, sensitivity: 1.0, specificity: 0.88). We report the first data demonstrating the presence of ANRIL and its transcript variants expressions in the AT and PBMCs of CAD patients. circANRIL having a synergetic effect with ANRIL plays a protective role in CAD pathogenesis. Therefore, altered circANRIL expression may become a potential diagnostic transcriptional biomarker for early CAD diagnosis.

## 1 INTRODUCTION

Coronary artery disease (CAD) is a complex disease and it is the most common cardiovascular condition.^1^ The risk of developing CAD is related to the interplay of genetic, lifestyle and environmental factors. Regional body adipose tissue (AT) distribution, rather than total body adiposity, is a well‐known marker for cardiovascular risk.^2^ In particular, visceral adipose tissue (VAT), including epicardial adipose tissues (EAT) and mediastinal (MAT), has been studied in the last decade in terms of their relationship with CAD. EAT is the most studied cardiac AT and it has gained increased attention due to its location between the visceral pericardium and the heart, in addition to the fact that it is in contact with the coronary vessels. Recent studies have shown that EAT has paracrine or endocrine activities, it can secrete inflammatory factors and adipocytokines, and it has the ability to release and uptake free fatty acids, which plays a vital role in the development of CAD.^3^ Moreover, studies have shown that the volume of EAT is associated with the incidence of CAD.^4^, ^5^, ^6^, ^7^ Another cardiac AT is MAT, which is situated outside the pericardium that encloses the heart and the relationship between MAT and the development of CAD has been demonstrated and a study reported that the increased volume of MAT was associated with an enhanced CAD risk profile.^8^ Also, the volume of MAT was determined to be positively correlated with plasma triacylglycerol and C‐reactive protein, which are well‐known risk factors and play a role in the progression of the disease.^9^ Furthermore, diseases associated with fat depots such as CAD are related with excessive accumulation of subcutaneous adipose tissue (SAT), while an increase in the number of adipocytes in the SAT is also accompanied by the presence of metabolic disorders.^10^

As with most complex diseases, genetics plays an important role in the development of CAD. Genome‐wide association studies (GWAS) have identified a new susceptibility region located on human chromosome 9p21.3 (Chr9p21.3) containing multiple single nucleotide polymorphisms (SNPs) associated with CAD.^11^, ^12^, ^13^, ^14^ The nearest genes, approximately 100 kilobases (kb) away from the core CAD region, are two tumour suppressor genes (cyclin‐dependent kinase inhibitors) CDKN2A and CDKN2B that play an important role in cell cycle regulation, apoptosis, aging and inflammation, which are processes strongly involved in atherogenesis. From a functional perspective, CDKN2A and CDKN2B are functional candidate genes that are potentially implicated in the pathogenesis of atherosclerosis.^15^, ^16^ Antisense non‐coding RNA (ncRNA) in the *INK4* locus (*ANRIL ENSG00000240498*) is another gene located at the same locus gene, which spans a region of almost 126.3 kb and *ANRIL* overlaps at its 5′ end with *CDKN2B* and it has a role in the epigenetic regulation of the expression of adjacent protein‐coding genes, including *MTAP*, *CDKN2A* and *CDKN2B* through multiple mechanisms.^17^, ^18^ Recently, it was revealed that the risk locus could be responsible for regulating *ANRIL* expression to a certain extent, while 9p21.3 risk locus could also have an impact on *CDKN2A* and *CDKN2B* expression through *ANRIL* expression.^19^, ^20^ Another study demonstrated that deletion of the targeted 9p21.3 risk locus of orthologous *ANRIL* interval in mice reduced the expression of *CDKN2A* and *CDKN2B* in the heart and led to excessive proliferation of vascular cells that contribute to the development of atherosclerosis.^21^ Several investigations have suggested that transcript variants of *ANRIL*, not only in linear transcript variants but also in circular transcript variants, could also be responsible for the development of CAD.^22^, ^23^, ^24^, ^25^ The most studied linear transcript variants of *ANRIL* with CAD are as follows; the longest *ANRIL* transcript variant: *NR003529* (*ENST00000428597.6*), and two shorter variants of *ANRIL*; *DQ485454* (*ENST00000580576.6*) and *EU741058* (*ENST00000455933.7*). Recently, *EU741058* and *NR003529* transcripts were found to be significantly increased in peripheral blood mononuclear cells (PBMCs) and atherosclerotic plaque tissue from CAD patients compared to healthy individuals. A newly discovered circular transcript variant of *ANRIL*, circular antisense ncRNA in the *INK4* locus (*circANRIL* or *hsa_circ_0008574*), consists of exons 5, 6 and 7, where exon 7 is non‐canonically spliced to exon 5. The *circANRIL* was found in many different cell lines and in many primary cell types, including vascular smooth muscle cells, macrophages, heart and vascular tissue.^24^, ^25^ Burd et al. found that *circANRIL* expression is associated with atherosclerotic risk.^25^ Another study by Holdt et al. speculated that *circANRIL* is a prototype of a circRNA regulating ribosome biogenesis and conferring atheroprotection, and in the same study, they suggested that *circANRIL* remains a potential therapeutic target for the treatment of atherosclerosis.^24^ Taken together, observations from all studies conducted thus far emphasize the importance of *ANRIL* and/or transcript variants of *ANRIL* in the mechanism mediating the 9p21.3 association, and suggest that *ANRIL* could be a possible candidate gene of CAD at the 9p21.3 risk locus.

The aim of the study was first to assess whether the AT and PBMCs expressions of *ANRIL* and *ANRIL* transcript variants were associated with CAD susceptibility, and to investigate the impact of 9p21.3 locus variations on the expression of *ANRIL* and transcript variants in PBMCs in AT in Tanzanian CAD patients.

## 2 MATERIALS AND METHODS

### 2.1 Subjects

This study was performed at Jakaya Kikwete Cardiac Institute, Dar es Salaam, Tanzania. Participants enrolled in the study were selected among patients admitted to the cardiology department outpatient clinic and patients admitted to the cardiology department for surgery. A total of 420 subjects including 200 CAD patients (175 patients with CAD from the outpatient clinic and 25 patients undergoing coronary artery bypass grafting [CABG] due to CAD) and 220 non‐CAD patients (195 patients without CAD from the outpatient clinic and 25 patients undergoing heart valve operation) were enrolled to investigate the association between the polymorphisms located on the 9p21.3 CAD risk locus and CAD susceptibility. Then, 25 CAD patients undergoing CABG and 25 patients undergoing heart valve operation were included to detect ANRIL and its transcript variants expressions in AT and PBMCs. CAD was defined as ≥50% luminal narrowing in at least one coronary artery (CA) for patients from the outpatient clinic and it was defined as ≥50% luminal narrowing in at least two CA for patients from the cardiology department surgery clinic. CAD classification is explained in the supplementary file. Only the patients with normal coronary angiogram were included in the non‐CAD group. All subjects enrolled in this study were of Tanzanian origin. Detailed information on the subjects' demographics, medical history, current medication and CAD risk factors was obtained through personal interviews. Oral antidiabetic and lipid lowering drugs that could interfere with gene expression were stopped 3 days before the operation and sample collection process. PBMCs and AT samples were obtained after overnight (12 h) fasting.

### 2.2 Biochemical measurements

Serum total cholesterol (TC) and high‐density lipoprotein cholesterol (HDL) and triglycerides (TG) were measured by routine enzymatic endpoint methods (Analyser A15 Biosystems, Philippines). Very low density lipoprotein cholesterol (VLDL) and LDL were calculated in keeping with Friedewald's Formula.^26^

### 2.3 Tissue biopsies

Approximately 30–50 mg of EAT, MAT and SAT were collected during surgery in the form of biopsy. EAT samples were collected from the fat surrounding the heart, within the pericardium, and MAT samples were collected from the fat within the mediastinum, outside the pericardial sac. SAT samples were collected from the retrosternal region. All AT was then immediately frozen in liquid nitrogen and stored at −80°C prior to total RNA preparation.

### 2.4 DNA and RNA extraction

Genomic DNA was obtained from the peripheral blood leukocytes of all participants by the use of a MagnaPure DNA isolation robot (Roche, Germany). Total RNA from EAT, MAT, SAT and PBMCs was extracted with Trizol™ Reagent (Invitrogen, Carlsbad, CA, USA) according to the manufacturer's instructions. DNA and RNA quantity was determined using a NanoDrop™ 1000 Spectrophotometer (Thermo Scientific, Wilmington, Delaware USA).

### 2.5 Genotyping

SNPs rs10757274, rs2383207, rs2383206, rs10811656 and rs10757278 in all participants were detected with the use of LightSNiP typing assays (TIB MolBiol, Berlin, Germany), employing quantitative real‐time polymerase chain reaction (QRT‐PCR) amplifications with melting curve analysis. The reactions were performed on a LightCycler®480 II Real‐Time PCR system (Roche‐Germany), following the recommendations of the manufacturer.

### 2.6 Gene expression analysis by quantitative real‐time PCR (QRT‐PCR)

The expression levels of the genes (*ANRIL*, *circANRIL*, *NR003529*, *EU741058*, *DQ485454* and *β‐ACTIN* as a housekeeping gene) were detected by qRT‐PCR using a LightCycler®480 II Real‐Time PCR system (Roche, Germany). After 1 μg of RNA was reverse transcribed using a Revert Aid First Strand cDNA Synthesis Kit (Fermentas, Canada), cDNA synthesis and expression analysis for *circANRIL* was performed as described by Vromman et al.^27^ Primers and probes designed using the soft‐ware Universal Probe Library (Table S1) and primers synthesized by IDT (Skokie‐USA) and probes synthesized by Roche Applied Science (Roche‐Germany).

### 2.7 Data analysis

Statistical analysis was performed using SPSS software (Statistical Package for the Social Sciences 25.0, SPSS Inc, Chicago, IL, USA). Quantitative variables were expressed as mean ± standard deviation (SD), and qualitative variables were expressed as percentages. The allelic frequencies of these polymorphisms between the non‐CAD and CAD patients and the demographic characteristics between the groups were estimated for categorical variables were compared using chi‐squared test. Hardy–Weinberg equilibrium (HWE) was assessed by Fischer's exact test. The gene expression data were obtained as Cycle Threshold (CT) values. The expression of each gene was compared between depots using the 2^ΔΔCT^method. The differences between normal and non‐normal distributed continuous variables were compared using the Student's *t*‐test and Mann–Whitney *U* test, respectively. To evaluate differences between groups, the data underwent log transformation to satisfy ANOVA criteria and then subjected to one‐way ANOVA with Tukey's post‐hoc analysis. The correlations between the gene expression and risk factors of CAD were evaluated with the Spearman correlation test. The statistical software package MedCalc Statistical Software (version 16.2, Ostend, Belgium) was used for multiple logistic regression analysis and receiver operating characteristic (ROC) analysis to examine expressions of target genes in the PBMCs can be used as a biomarker for CAD. A logistic regression classifier implemented in the scikit‐learn library^28^ was used to perform leave‐one‐out cross validation and to calculate the *p*‐value using the method proposed by DeLong et al.^29^ Normalized coefficient magnitudes were used as a means of calculating feature importance. Figures were generated in Python using the matplotlib library.^30^ Statistical significance was taken as *p* < 0.05.

## 3 RESULTS

### 3.1 Clinical and anthropometric characteristics of the study population

The clinical and anthropometric characteristics of the study group are presented in Table 1. When the lipid profiles were compared, serum TG, TC, LDL were higher in all CAD patients than in the non‐CAD patients (*p* < 0.05, respectively), whereas serum HDL levels were significantly higher among non‐CAD patients *(p* < 0.05). The average fasting plasma glucose levels of the CAD patients were significantly higher than those of non‐CAD patients (*p* < 0.05). CAD patients had higher levels of systolic blood pressure and diastolic blood pressure. There was also a higher prevalence of obesity, hypertension, diabetes, hyperlipidaemia and smokers in CAD patients compared to non‐CAD patients (*p* < 0.05). In addition, CAD patients were predominantly male (76%). Hypertension was the more commonly associated clinical condition in CAD patients (92%), while the least common one was determined to be hyperlipidaemia (56%).

**TABLE 1:** Baseline characteristics of the study groups.

Variables	CAD (*n* = 200)	Non‐CAD (*n* = 220)	*p*‐value^a^
Age (years)	60.04 ± 7.67	58.64 ± 10.27	0.580
Sex/male (%)	19 (76%)	10(40%)	***0.010***
Weight (kg)	84.56 ± 11.58	67.52 ± 14.56	***0.001***
Height (m)	1.60 ± 0.088	1.65 ± 0.073	***0.291***
BMI (kg/m^2^)	32.36 ± 4.70	23.84 ± 3.98	***0.001***
Systolic BP (mmHg)	133.88 ± 28.89	122.64 ± 24.56	***0.037***
Diastolic BP (mmHg)	91.40 ± 15.54	77.08 ± 16.33	***0.003***
Glucose (mmol/L)	8.01 ± 2.14	4.13 ± 0.78	***0.001***
Cholesterol (mmol/L)	5.88 ± 0.95	3.95 ± 0.72	***0.001***
HDL (mmol/L)	0.84 ± 0.28	1.36 ± 0.30	***0.001***
LDL (mol/L)	3.97 ± 0.76	2.56 ± 0.66	***0.001***
VLDL (mmol/L)	1.19 ± 0.44	0.52 ± 0.28	***0.001***
TG (mmol/L)	2.63 ± 1.31	0.98 ± 0.41	***0.001***
Obesity (%)	18 (72%)	0	***0.001***
Hypertension (%)	23 (92%)	11 (44%)	***0.001***
Diabetes (%)	17 (68%)	1 (4%)	***0.001***
Hyperlipidaemia (%)	14 (56%)	0	***0.001***
Smoking (%)	17 (68%)	7 (28%)	***0.005***

*Note*: Values are presented as mean ± SD.

Bold and italic values are indicates statistical significant value (*p* < 0.05).

^a^ Comparisons of differences between mean values of two groups unpaired Student's *t*‐test was used.

### 3.2 Allele and genotypic association of different SNPs

The allele and genotype frequencies of five SNPs (rs10757274, rs2383207, rs2383206, rs10811656 and rs10757278) were analysed for all participants including 200 CAD patients and 220 non‐CAD patients and the results are presented in Table S2. All SNPs were at HWE in both groups (*p* > 0.05). Significant differences were observed in the genotype and allele frequencies of rs10757274, rs2383206, rs10811656 and rs10757278 variants between CAD and non‐CAD patients (*p* < 0.005). The genotype frequencies of rs10757274, rs2383206, rs10811656 and rs10757278 SNPs remained significant when analysed in the subgroup including 25 CAD patients undergoing CABG surgery and 25 non‐CAD patients undergoing heart valve surgery (Table 2). Furthermore, the risk alleles rs10757274 G allele, rs2383206 G allele, rs10811656 T allele and rs10757278 G allele were found to be statistically significant in CAD patients compared to non‐CAD patients of the subgroup.

**TABLE 2:** The genotypic and allelic frequency distributions of SNPs on chromosome 9p21.3 in the subgroup.

SNP	Genotypic frequencies *n* (%)			*p*‐value^a^	Allelic frequencies			*X* ^2^	OR/CI(95%)	*p*‐value
	Genotype	CAD (*n* = 25)	Non‐CAD (*n* = 25)		Allele	CAD (*n* = 25)	Non‐CAD (*n* = 25)			
rs10757274	AA	3 (12)	17 (68)							
	AG	16 (64)	7 (28)	*0.001*	A/G	0.44/0.56	0.82/0.18	15.49	5.79/2.32–14.43	*0.001*
	GG	6 (24)	1 (4)							
rs2383207	AA	3 (12)	12 (48)							
	AG	13 (52)	2 (8)	0.563	A/G	0.38/0.62	0.52/0.48	1.98	1.76/0.79–3.91	0.159
	GG	9 (36)	11 (44)							
rs2383206	AA	3 (12)	14 (56)							
	AG	13 (52)	7 (28)	*0.001*	A/G	0.38/0.62	0.70/0.30	10.31	3.80/1.65–8.74	*0.001*
	GG	9 (36)	4 (16)							
rs10811656	CC	6 (24)	19 (76)							
	CT	11 (44)	4 (16)	*0.002*	C/T	0.46/0.54	0.84/0.16	15.87	6.16/2.41–15.75	*0.001*
	TT	8 (32)	2 (8)							
rs10757278	AA	7 (28)	19 (76)							
	AG	12 (48)	5 (16)	*0.001*	A/G	0.52/0.48	0.86/0.14	13.51	5.67/2.14–14.99	*0.001*
	GG	6 (24)	1 (8)							

*Note*: The subgroup includes 25 CAD patients undergoing CABG and 25 non‐CAD patients undergoing valve placement.

Abbreviations: CI, confidence interval; OR, odd ratio.

Bold and italic values are indicates statistical significant value (*p* < 0.05).

^a^ The genotypic and allelic frequency distributions of polymorphisms between the groups were compared using x^2^ and HWE test. In all cases differences were considered significant at *p* < 0.05.

### 3.3 ANRIL and ANRIL splice variants expression levels in AT and PBMCs

**FIGURE 1:**
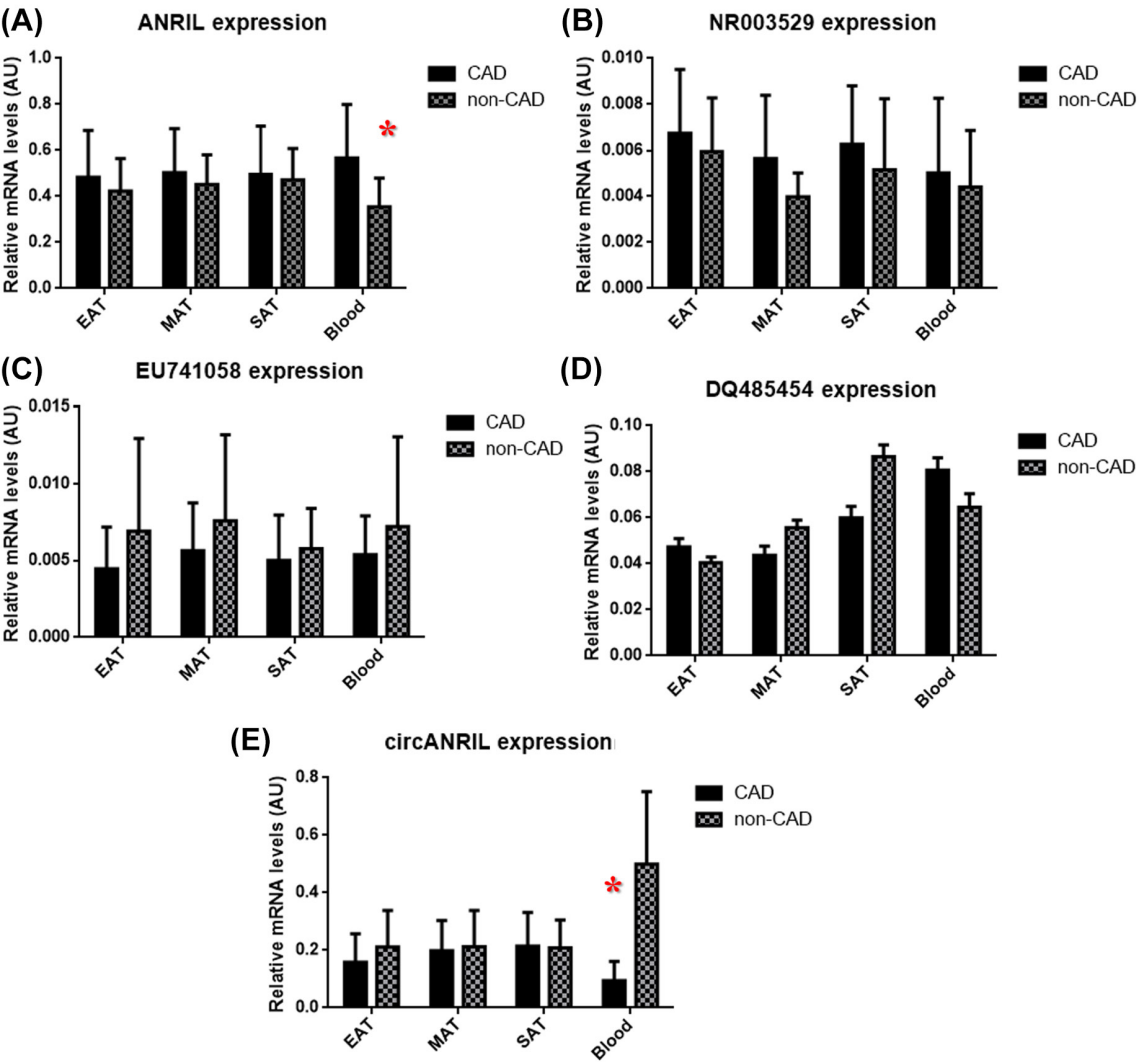
Relative expression levels of ANRIL, NR003529, EU741058, DQ485454 and cicrANRIL in EAT, MAT, SAT and in PBMCs among subgroup includes 25 CAD patients undergoing CABG and 25 non‐CAD patients undergoing valve placement. (A) Relative expression levels of ANRIL in CAD and non‐CAD patients; (B) Relative expression levels of NR003529 in CAD and non‐CAD patients; (C) Relative expression levels of EU741058 in CAD and non‐CAD patients; (D) Relative expression levels of DQ485454 in CAD and non‐CAD patients and (E) Relative expression levels of circANRIL in CAD and non‐CAD patients. AU, arbitrary unit; PDMC, peripheral blood mononuclear cells. *p < 0.05.

**FIGURE 2:**
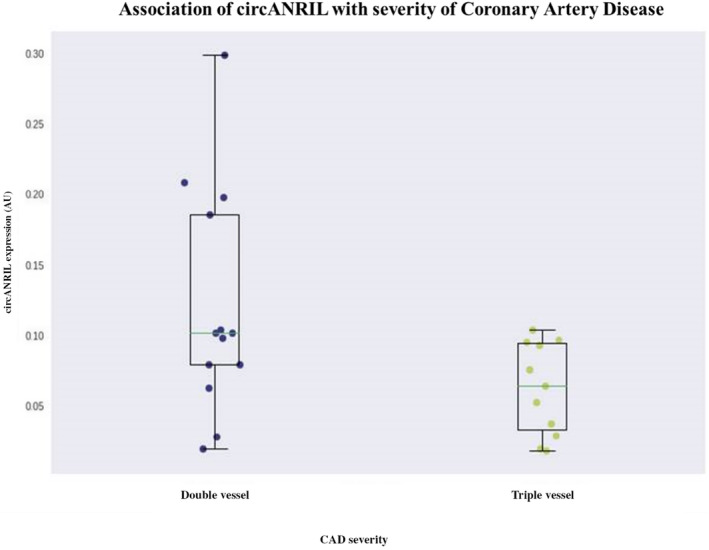
Association of expression levels of circANRIL with severity of coronary artery disease. The analyses were done in subgroup CAD patients that were divided according to the severity of the disease.

For expression analysis, we included 25 CAD patients undergoing CABG and 25 non‐CAD patients undergoing valve replacement. The expression levels of *ANRIL, circANRIL, NR003529, EU741058* and *DQ485454* were studied in EAT, MAT, SAT and PBMCs (Figure 1). *ANRIL* expression levels were significantly up‐regulated in PBMCs of the CAD patients compared to non‐CAD patients (fold change = 1.6, *p* < 0.001). Although *ANRIL* expression levels in EAT, MAT and SAT were found to be increased in CAD patients compared to non‐CAD patients, the differences were not significant (Figure 1A). *circANRIL* was significantly down‐regulated in PBMCs of CAD patients compared to non‐CAD patients (fold change = 5.3, *p* < 0.001) (Figure 1E). The associations of *ANRIL* and *circANRIL* expressions with the CAD severity (double stenotic vessels disease [*n* = 13] versus triple stenotic vessels disease [*n* = 12]) were also evaluated. A statistically significant difference in the expression levels of *circANRIL* was determined between the two groups (*p* < 0.05) (Figure 2). Moreover, the expression levels of *EU741058* in PBMCs was down‐regulated in CAD patients compared to non‐CAD (fold change ≈0.8), but the difference was not significant.

### 3.4 Associations between 9p21.3 risk locus genotypes and ANRIL and ANRIL transcript expression levels

To better understand the relationship between *ANRIL* and CAD, we next evaluated the potential effects of 9p21.3 risk locus SNPs on the expression levels of *ANRIL* and *ANRIL* transcript variants in PBMCs and AT of the study subgroups. The expression levels of *ANRIL* in PBMCs were significantly higher in the risk genotype carriers of rs10757278 and rs10811656 (GA and GG for rs10757278 and CT and TT for rs10811656) in CAD patients compared to wild type carriers (*p* = 0.004 and *p* = 0.013, respectively). Also, the expression levels of *ANRIL* transcript variants *NR003529* and *EU741058* in EAT, MAT and PBMCs were significantly higher in CAD patients carrying the risk genotype of rs10757278 and rs10811656 compared to wild type carriers, while there was no difference in *DQ485454* (*p* = 0.001, *p* = 0.007, *p* = 0.028, *p* = 0.006, *p* = 0.002, *p* = 0.019 and *p* > 0.05, respectively) (Figure S1). However, the expression levels of *circANRIL* in PBMCs were significantly down‐regulated in rs10757278 and rs10811656 risk genotype carriers compared to wild type carriers (*p* = 0.001 and *p* = 0.01, respectively).

### 3.5 Impacts of CAD risk factors on the expression levels of ANRIL transcripts

The correlation between the expression levels of candidate genes and risk factors of CAD were analysed using the Spearman correlation test (Figure S2). *ANRIL* expression in PBMCs was positively correlated with BMI, glucose level, TC, TG and LDL (*r* = 0.362, *p* = 0.01; *r* = 0.325, *p* = 0.021; *r* = 0.323, *p* = 0.02; *r* = 0.444, *p* = 0.001 and *r* = 0.460, *p* = 0.001, respectively) but negatively associated with HDL (r = 0.304, *p* = 0.032). The *circANRIL* expression levels in PBMCs were negatively correlated with BMI, glucose level, TC, TG, LDL, Systolic BP and Diastolic BP (*r* = 0.531, *p* = 0.001; *r* = 0.547, *p* = 0.001; *r* = 0.599, *p* = 0.001; *r* = 0.558, *p* = 0.001; *r* = 0.535, *p* = 0.001; *r* = 0.363, *p* = 0.009; and *r* = 0.469, *p* = 0.001, respectively) but positively associated with HDL (*r* = 0.583, *p* = 0.001). *ANRIL* and *circANRIL* were co‐regulated with most of the risk factors of CAD such as lipid levels, blood pressure and glucose levels. These positive and negative correlation results with the risk factors suggest that *ANRIL* and *circANRIL* expressions regulate the risk factors leading to CAD development and *ANRIL* and *circANRIL* may serve as indicator genes in CAD patients.

### 3.6 Future importance of the variables

**FIGURE 3:**
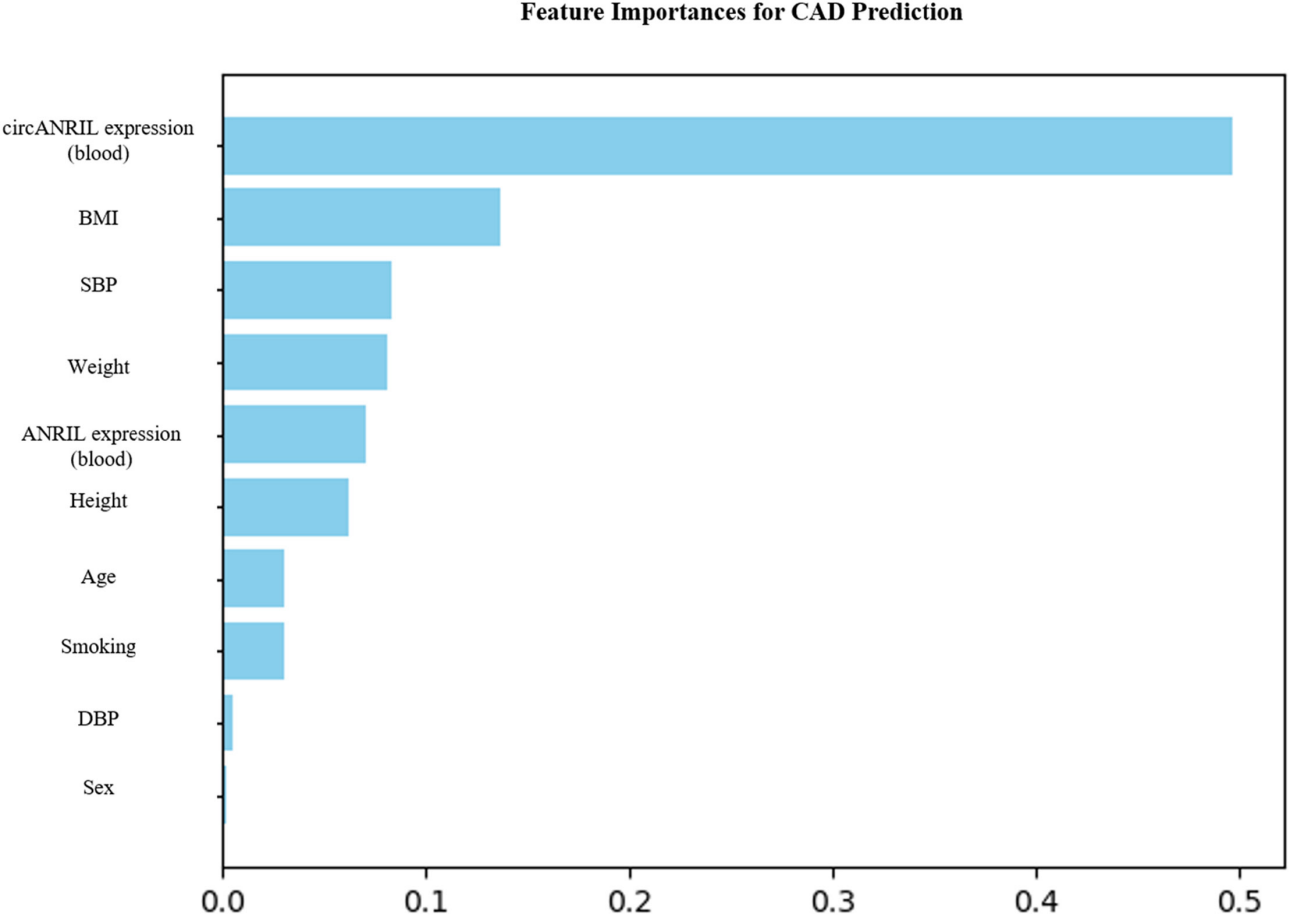
Feature importance analysis of included variables obtained from a tuned random forest model in Tanzanian CAD patients. BMI, body mass index; DBP, diastolic blood pressure; SBP, systolic blood pressure.

Machine learning (ML) is a highly effective method for disease prediction using ML techniques. It is able to capture the complex interactions between predictors and outcomes during the data process, and could provide a new and novel discernment towards the disease.^31^ Recent studies show that random forest (RF) is the most efficient algorithm for the prediction of CAD and it consistently improves the accuracy of the prediction system.^32^, ^33^ We measured the importance of different features for the risk factors of CAD together with the expression levels of *ANRIL* and its transcript variants by the mean decrease impurity (Gini importance) of all decision trees in a tuned RF model. The importance of included variables obtained from the tuned RF model is presented in Figure 3. As expected, age, systolic BP, BMI and smoking were among the top risk factors. In addition, we observed that expression levels of *ANRIL* and *circANRIL* in PBMCs were also among the top risk factors.

### 3.7 Improvement in the diagnostic value

**FIGURE 4:**
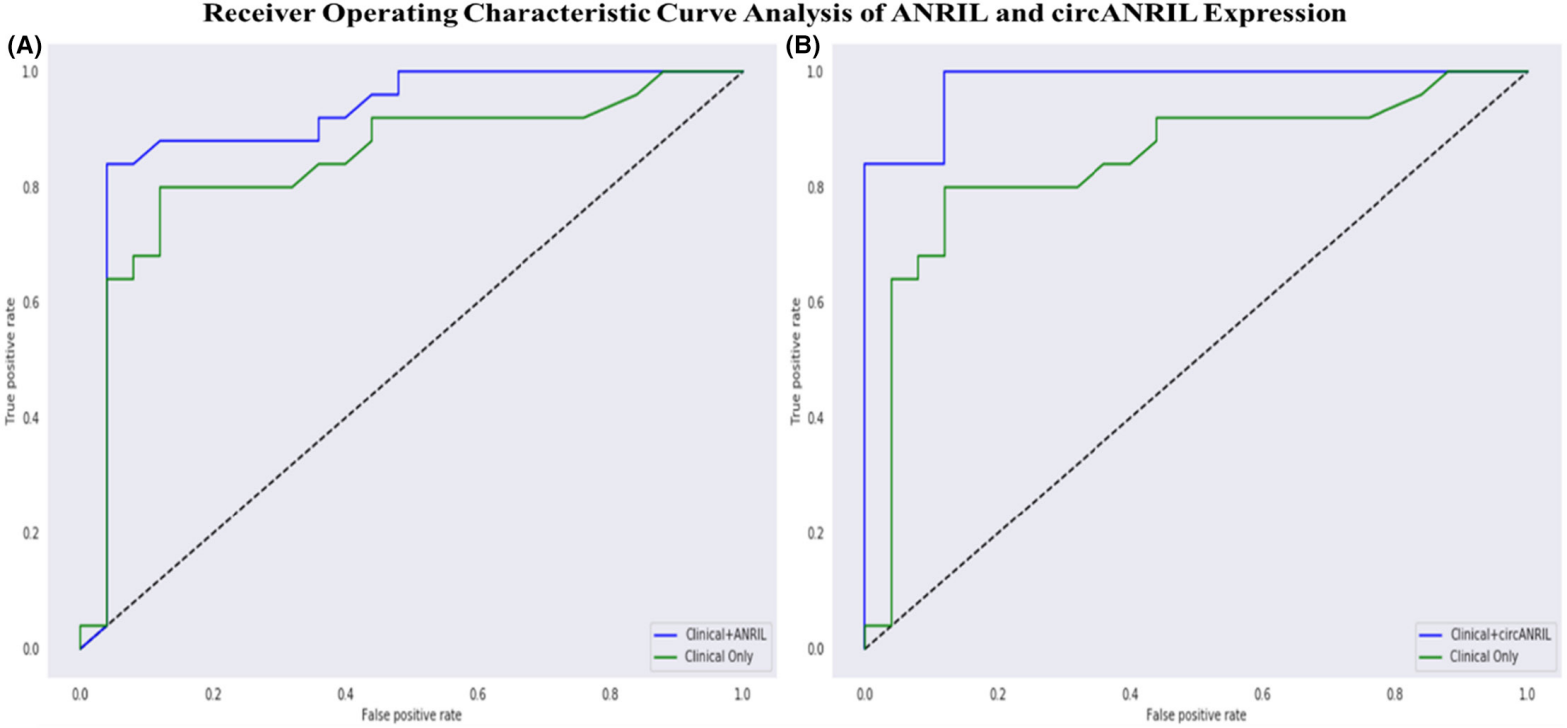
ROC curve analyses of the diagnostic value of expression levels of *ANRIL* and *circANRIL*. ROC curve comparisons between models. (A) Clinical model and clinical + ANRIL expression model, (B) clinical model and clinical + circANRIL expression model. AUC, area under curve; ROC, receiver operating characteristic.

Multivariate logistic regression analyses revealed that weight (OR = 0.91, 95%CI = 0.85–0.96), height (OR = 3.54, 95%CI = 1.60–7.81), BMI (OR = 0.61, 95%CI = 0.46–0.80), systolic BP (OR = 0.98, 95%CI = 0.96–1.0), smoking (OR = 5.46, 95%CI = 1.62–18.35), diastolic BP (OR = 0.95, 95%CI = 0.90–0.99), sex (OR = 0.22, 95%CI = 0.06–0.71) and the expression levels of *ANRIL* (OR = 2.05, 95%CI = 1.74–2.35) and *circANRIL* (OR = 13.63, 95%CI = 3.74–49.60) in PBMCs were potential transcriptional biomarkers for CAD. To test comparisons of the diagnostic value of expression levels of *ANRIL* and *circANRIL* to the top risk factors which are clinical features and observed in the RF model in the development of CAD, ROC curve analysis was performed and the area under curve (AUC) was calculated. Three models for CAD prediction based on clinical features and expressions of *ANRIL* and *circANRIL* were built. The first model (clinical model) consisted of CAD risk factors: weight, height, BMI, systolic BP, diastolic BP, smoking and age. The second model (clinical + *ANRIL* expression model) consisted of the clinical model and *ANRIL* expression, and in the last model (clinical + *circANRIL* expression model), *circANRIL* expression was included in the clinical model. The AUC value was determined to be 0.844 for the clinical model (95% CI: 0.724–0.963, optimal cut‐off: 0.55, specificity: 0.88, sensitivity: 0.80), while the introduction of *ANRIL* expression increased the AUC from 0.844 to 0.912 (95% CI 0.821–1.0, optimal cut‐off:0.61, specificity:0.96, sensitivity:0.84, *p* = 0.02) (Figure 4A), while the *circANRIL* expression into the clinical model including the CAD risk factors (age, weight, height, BMI, systolic BP and smoking) the AUC significantly increased from 0.844 to 0.980 (95%CI 0.953–1.0, optimal cut‐off:0.33, specificity:0.88, sensitivity: 1.0, *p = 0.009*) (Figure 4B). Finally, ROC analyses suggested that the detection of *ANRIL* expression and *circANRIL* expression together with risk factors of CAD exhibited a higher diagnostic performance compared with the detection of risk factors only. This result implies that the combination of *ANRIL* and *circANRIL* expressions in PBMCs has significant potential to be a sensitive and reliable transcriptional biomarker that possibly has a higher diagnostic value for CAD.

## 4 DISCUSSION

While a big part of the human genome has already been transcribed, only ≈2% of the genome appear to be protein‐coding, the rest being deemed ‘non‐coding’. Recent studies have demonstrated that the human genome is prevalently transcribed and produce many thousands of ncRNAs that play a vital role in cellular processes, and the identification of the role of ncRNAs in the disease process could increase our understanding of the pathogenesis of complex diseases.^34^ Accumulating studies indicate that several long ncRNAs (lncRNAs), which are a member of the ncRNAs, play important roles as key regulators of gene regulation in the development of cardiovascular diseases.^35^ *ANRIL* is one of the most important antisense transcripts lncRNA, receiving widespread attention as a potentially novel biological regulator. Many roles have been attributed to *ANRIL* including DNA damage response, DNA repair, epigenetic regulation of *CDKN2A/B*, controlling the cell cycle checkpoints, apoptosis and RNA splicing.^36^ A growing number of recent studies confirm that *ANRIL* plays pivotal roles in diverse physiological and pathological processes in CAD.^17^, ^19^, ^20^, ^21^, ^22^, ^23^, ^24^, ^25^, ^37^ We herein aimed to ascertain whether the ANRIL and ANRIL transcript variants were involved in the manifestation of CAD susceptibility and where ANRIL transcripts are associated with the 9p21.3 CAD risk in the Tanzanian patients. In the present study, the expression of ANRIL and different transcript variants of ANRIL in PBMCs and in various ATs was reported for the first time.

We reported differential expressions of ANRIL and its transcript variants in CAD patients compared to non‐CAD patients. ANRIL expression levels were significantly increased in the PBMCs of CAD patients, but no statistically significant expression was observed in AT for ANRIL also linear transcripts. On the other hand, one of the main findings of our study was that circANRIL expression was decreased in PBMCs of CAD patients compared with non‐CAD patients, and it well‐discriminated CAD patients from non‐CAD patients, and the circANRIL level was associated with increased CAD severity. Our study demonstrated that up‐regulated ANRIL expression could possibly be involved in CAD pathogenesis. This is in concordance with other studies that investigated the association of ANRIL expression and CAD in different populations.^38^, ^39^ Moreover, it was demonstrated that increased expression of ANRIL led to increased inflammation, CAD risk, severity and poor prognosis of CAD.^40^, ^41^, ^42^ ANRIL expression was also shown to be associated with inflammation cytokines MCP1 and IL‐10^42^ and the knockdown of ANRIL significantly promoted cell proliferation and tubule formation and inhibited inflammatory activation and apoptosis of human umbilical vein endothelial cells.^42^ In contrast, in the study of Yang et al. no significant association was reported between CAD and ANRIL expression in the Chinese Han population.^43^

Furthermore, we observed a significant down‐regulation of *circANRIL* expression in CAD patients compared to non‐CAD patients, and according to the comparison of circANRIL expression according to the severity of CAD, we found that triple stenotic vessels disease CAD patients had lower expression levels of circANRIL compared to double stenotic vessels disease CAD patients. We therefore suggest that high expression of *circANRIL* may play an atheroprotective role in CAD pathogenesis, which would indicate a synergetic effect between *circANRIL* and *ANRIL*. Holdt et al. also suggested that *circANRIL* has a protective role against atherosclerosis.^24^ Moreover, recent studies have shown that increased *circANRIL* expression in human primary smooth muscle cells and vascular endothelial cells in a rat model of CAD could induce apoptosis and inhibit the proliferation; therefore, increased *circANRIL* expression could decrease the susceptibility of CAD.^44^, ^45^ However, we failed to detect the same significance of *ANRIL* and *circANRIL* expression levels in AT. One potential explanation for this result could be that *ANRIL* and *circANRIL* could be differentially expressed in AT and PBMCs. We also found decreased expression levels of *EU741058* in PBMCs of CAD patients compared to non‐CAD patients, but the differences only had borderline statistical significance. Several previous studies have reported that *EU741058* variant down‐regulated in CAD patients and these results are in agreement with our findings.^43^, ^46^

Further analysis revealed that rs10757278 and rs10811656 altered the expression levels of *ANRIL* and *ANRIL* transcript variants, not only in PBMCs but also in AT. This result emphasized that rs10757278 and rs10811656 could control the expression levels of *ANRIL* and *ANRIL* transcript variants. The *circANRIL* expression levels in PBMCs were significantly down‐regulated in the CAD risk genotype carriers of rs10757278 (GA and GG) and rs10811656 (CT and TT) compared to wild type carriers. Also, the risk genotypes carriers of rs10757278 (GA and GG) and rs10811656 (CT and TT) had significantly higher *ANRIL* expression in the EAT, MAT and in PBMCs of CAD patients. A recent study by Liu et al. showed that the expression of *ANRIL* significantly decreased in rs10757278 risk allele carriers in the T cells of 170 healthy individuals.^47^ A potential explanation for the diverse results between the research by Liu et al., and our study might be that the samples they used were T‐cells from healthy individuals, whereas in our research, the samples were from EAT, MAT and PBMCs of patients suffering from CAD.^47^ Also, the expression levels of *NR003529* and *EU741058* in EAT, MAT and in PBMCs were statistically significantly higher in the CAD patients carrying the risk genotype of rs10757278 and rs10811656 compared to wild type carriers, but the expression of *DQ485454* remained unaffected. This finding indicated that *NR003529* and *EU741058* were abundantly transcribed in CAD risk genotype carriers leading to the development of CAD.

It is well known that EAT is an important component of VAT due to its contiguity to the CA and the effects of endocrine and paracrine activity secreting pro‐inflammatory and anti‐inflammatory cytokines and chemokines, and it has therefore been suggested to influence coronary atherosclerosis development.^48^, ^49^ In recent years, MAT has also gained increasing attention due its contribution to the development of atherosclerosis.^50^, ^51^ Our results also demonstrated the clinical importance of both EAT and MAT in CAD.

Another finding in our study was the increased expression levels of *circANRIL* in PBMCs. which was also associated with reduced severity of CAD. The *circANRIL* expression was statistically significantly decreased in triple stenotic vessels disease compared to double stenotic vessels disease (2‐fold). A previous study reported that *EU741058* and *NR00359* expressions were pre‐dominantly associated with the severity of CAD.^22^ Although we could not find any association between *EU741058* and *NR00359* expression and CAD severity, we successfully showed that *circANRIL* expression was directly associated with the severity of CAD.

In clinical practice, the increased levels of glucose, TC, TG, LDL and BMI, decreased HDL levels and also hypertension are consistently attributed as major risk factors for CAD. In our study, we observed that these risk factors of CAD were correlated with *ANRIL* and *circANRIL* expression levels in the blood. This co‐regulation suggests that *ANRIL* and *circANRIL* regulate the risk factors leading to CAD development and they may serve as early disease indicators in CAD patients.

Although the CAD mortality rate is high, the chance of survival is higher if the diagnosis is made sufficiently early in an accurate and efficient way. Therefore, experts have attempted to identify a method of accurately predicting CAD at an early stage by using new statistical techniques, such as data mining, which could help to identify the risk models of the disease, as well as to recognize the disease patterns and the influencing factors. The feature importance selection methods provide the opportunity to increase the accuracy of the prediction of disease compared to traditional methods of classification. In particular, the combination of the feature importance selection algorithm such as RF, which has better accuracy for CAD prediction than other algorithms, is necessary for the efficient prediction of CAD.^33^, ^52^ We examined the importance of different features for the risk factors of CAD together with the expression levels of ANRIL and its transcript variants and we observed that age, systolic BP, BMI and smoking were among the top risk factors as we expected. In addition, the expression levels of ANRIL and circANRIL in PBMCs were also among the top risk factors. The ROC analysis was conducted accordingly and the analyses showed that a good predictive value of circANRIL expression together with risk factors of CAD exhibited a higher diagnostic performance compared to CAD risk factors alone. The circANRIL expression levels in PBMCs may be a new and non‐invasive diagnostic tool for the diagnosis of CAD. As a screening tool in clinical practice, circANRIL has potential diagnostic value and is worthy of clinical promotion.^53^

**FIGURE 5:**
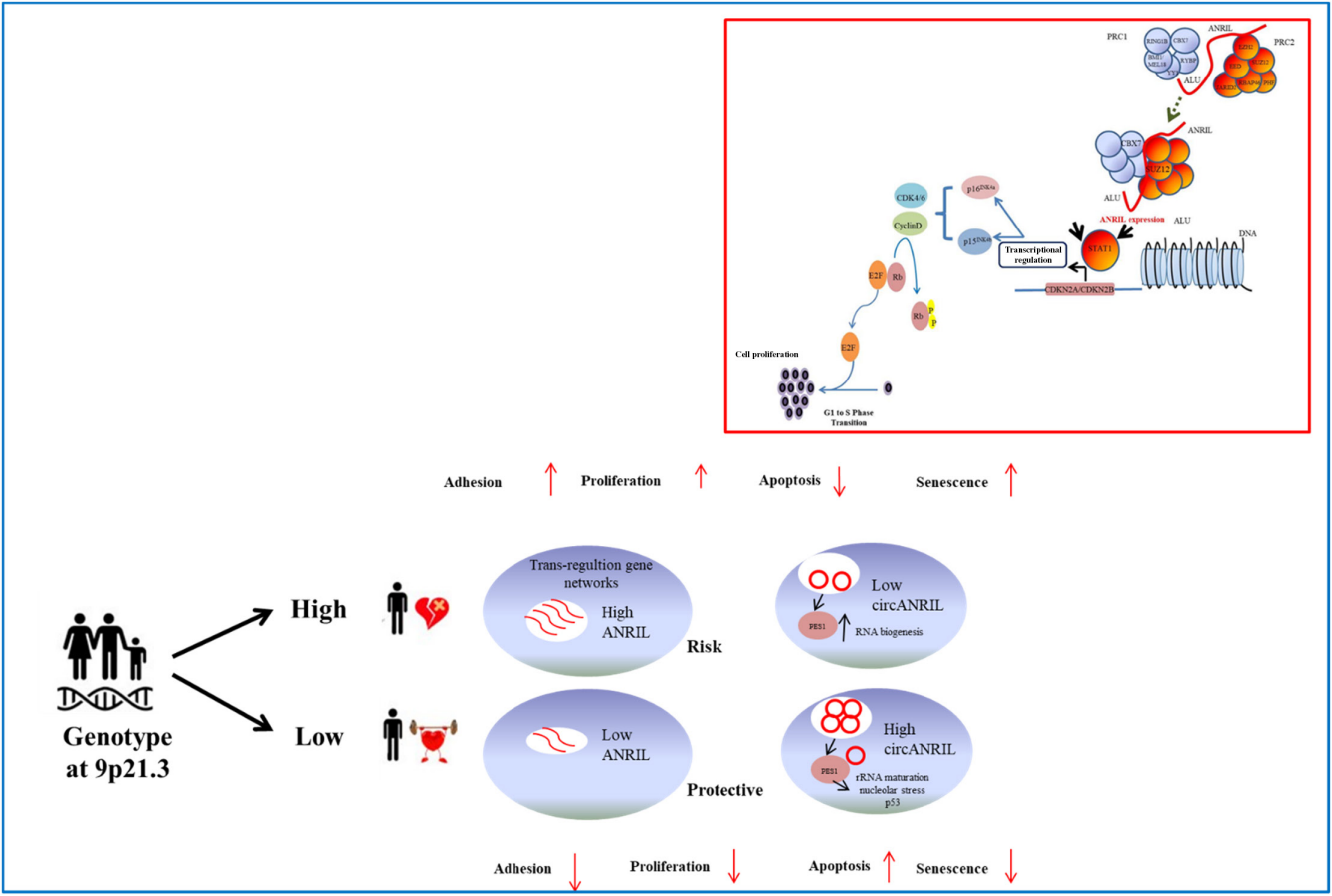
The possible effect mechanism of the genotype of 9p21.3of linear ANRIL and circANRIL functions according to our results. The risk alleles lead to up‐regulation of ANRIL and down‐regulation of circANRIL. Increased ANRIL expression regulated of the expression of adjacent protein‐coding genes, including CDKN2A and CDKN2B leading to pro‐atherogenic cell properties (increased cell adhesion, increased proliferation and decreased apoptosis) through CBX7 and SUZ12 function in the polycomb complex.^55^, ^56^ circANRIL binds PES1 protein and impairs ribosome biogenesis, leading to activation of p53 and a subsequent increase in apoptosis and a decrease in proliferative rate.^24^ PES1, pescadillo ribosomal biogenesis factor 1; CBX7, Chromobox homologue 7; p53, tumour protein 53; SUZ12, polycomb repressive complex 2 subunit.

ANRIL became one of the most popular lncRNA since the discovery of 9p21.3 region as a susceptibility region for CAD. The SNPs in the 9p21.3 region are located 4‐bp apart in the STAT1 binding site of the ANRIL gene.^54^ The risk SNPs could disrupt the binding site of STAT1 and this would in turn affect the expression of ANRIL, leading to increased enhancer activity through the following possible mechanism (Figure 5). It is well known that ANRIL regulates the expression of protein‐coding genes via a physically interaction with the specific site, including the Alu element to CBX7 and SUZ12 components of the Polycomb complex (PRC1 and PRC2). ANRIL acts as a pivotal regulator and affects the expression of CDKN2A and CDKN2B tumour suppressor genes in 9p21.3 locus through the same mechanism^55^, ^56^ (Figure 5). On the other hand, in an animal model, it was demonstrated that ANRIL upregulates vascular endothelial growth factor (VEGF) and activates the nuclear factor kappa B (NF‐κB) signalling pathway.^38^, ^57^ Moreover, the *circANRIL*, binds and negatively regulates pescadillo ribosomal biogenesis factor 1 (*PES1*) protein in the *PeBoW* complex, known as an essential complex to ribosome biogenesis in macrophages and vascular smooth muscle cells. Furthermore, this negative regulation induces *p53* activation and nucleolar stress, resulting in the inhibition of proliferation and induction of *p53* in proliferating cells^24^ (Figure 5). Taken together, our results emphasize that significant changes of *ANRIL* and *circANRIL* expression levels could be directly or indirectly influenced by the progression of atherosclerosis in CAD. *ANRIL* and *circANRIL* could be a feature as potential transcriptional biomarkers as well as therapeutic targets and they might also be a useful disease marker.

## 5 CONCLUSIONS

Our results revealed that *ANRIL* expression levels in PBMCs were significantly increased while *circANRIL* expression levels were significantly decreased in CAD patients when compared to non‐CAD patients. Additionally, *circANRIL* expression was found to be related with the severity of CAD. Both rs10757278 and rs10811656 SNPs were also determined to be associated with not only *ANRIL* but also transcript variants of *ANRIL* (*EU741058*, *NR003529* and *circANRIL*). Not last, the RF model and ROC analyses showed that measuring the expression levels of circANRIL could offer reliable and sensitive prognostic value and allow for early detection and better monitoring of treatment response or disease recurrence for CAD. Taken together, our findings can provide a new liquid transcriptional biomarker to be used in combination with clinical decision‐making that can improve the early diagnosis and treatment of cardiovascular diseases.

## AUTHOR CONTRIBUTIONS

**Gokce Akan:** Conceptualization (lead); data curation (lead); formal analysis (lead); funding acquisition (equal); investigation (lead); methodology (lead); project administration (equal); resources (equal); software (equal); supervision (equal); validation (equal); visualization (equal); writing – original draft (lead); writing – review and editing (equal). **Evarist Nyawawa:** Data curation (equal); writing – review and editing (equal). **Bashir Nyangasa:** Data curation (equal); writing – review and editing (equal). **Mehmet Kerem Turkcan:** Formal analysis (equal); software (equal); writing – review and editing (equal). **Erasto Mbugi:** Conceptualization (equal); project administration (equal); supervision (equal); writing – review and editing (equal). **Mohammed Janabi:** Conceptualization (equal); project administration (equal); supervision (equal); writing – review and editing (equal). **Fatmahan Atalar:** Conceptualization (equal); funding acquisition (equal); project administration (equal); supervision (equal); writing – review and editing (equal). A preprint has previously been published.^58^

## FUNDING INFORMATION

This research received no specific grant from any funding agency in the public, commercial, or not‐for‐profit sectors.

## CONFLICT OF INTEREST STATEMENT

The authors declare that they have no conflicts of interest.

## Supporting information


Data S1:

## Data Availability

The datasets generated during and/or analysed during the current study are available from the corresponding author on reasonable request.
