# Supplementary material for: Severity of coronary artery disease is associated with diminished circANRIL expression: A possible blood based transcriptional biomarker in East Africa
Source: J Cell Mol Med. 2023 Dec 27;28(3):e18093. doi: 10.1111/jcmm.18093 (PMC10844708; doi:10.1111/jcmm.18093)
Supplement: Supplementary file 1 — Data S1: [file JCMM-28-e18093-s001.docx]

**CAD classification**

CAD patients from cardiology department surgery clinic underwent bypass surgery due to at least two vessel disease according to the number of significantly affected vessels using the Coronary Artery Surgery Study classification. Body mass index (BMI) was calculated as a ratio of mass in kilogram divided by the square of height in meters. Diagnosis of hypertension was defined as resting blood pressure ≥ 140/90 mm Hg or current use of antihypertensive therapy. Diagnosis of diabetes was defined either by 1999 World Health Organization (WHO) criteria 11 or self-report of being previously diagnosed as diabetic. Diagnosis of dyslipidemia was defined as low-density lipoprotein (LDL) cholesterol ≥3 mmol/l or the use of hypolipidemic agents.

**Supplementary Table 1:** PCR primers and probes for quantitative analysis of gene expressions

|  | Forward Primer | Reverse Primer | Probe |
| --- | --- | --- | --- |
| ANRIL | actccaaagaaaccatcagagg | tgctcctattcccaaattc | #39 (cat. no. 0468797330001) |
| circANRIL | accgctgcactacactacct | tactcctcccccaccatctg | #47 (cat. no. 046880740001) |
| NR003529 | gaactcccaggctcaaacc | ggagggagcatgtctgtttc | #33 (cat. no. 04687663001) |
| EU741058 | tcaaaggagaattttcttggaaag | aagcagtactgactcgggaaag | #31 (cat. no. 04687647001) |
| DQ485454 | tcaaaggagaattttcttggaaag | aagcagtactgactcgggaaag | #31 (cat. no. 04687647001) |
| β-ACTIN | attggcaatgagcggttc | ggatgccacaggactcca | #11 (cat. no. 04685105001) |

**Supplementary Table 2:** The genotypic and allelic frequency distributions of SNPs on chromosome 9p21.3 in study groups

| **SNP Genotypic Frequencies n (%) P-Value Allelic Frequencies X^2^ OR/CI(95%) P-Value** |
| --- |
| **Genotype CAD(n=200) non-CAD (n=220) Allele CAD(n=200) non-CAD (n=220)** |
| **rs10757274**  **AA 73(36.5) 159(72.3)**  **AG 88(44) 53(24.1) *0.001* A/G 0.58/0.42 0.84/0.16 69.31 3.81/2.75-5.27 *0.001***  **GG 39(19.5) 8 (3.6)** |
| **rs2383207**  **AA 3(1.5) 8(3.6)**  **AG 38(19) 43(19.5) 0.379 A/G 0.11/0.89 0.13/0.87 1.13 1.25/0.82-1.90 0.287**  **GG 159(79.5) 169(76.8)** |
| **rs2383206**  **AA 44(22) 120(54.5)**  **AG 110(55) 79(35.9) *0.001* A/G 0.49/0.51 0.72/0.28 46.83 2.69/2.01-3.58 *0.001***  **GG 46(23) 21(9.6)** |
| **rs10811656**  **CC 40(20) 125(56.8)**  **CT 100(50) 75(34.1) *0.001* C/T 0.45/0.55 0.74/0.26 72.80 3.45/2.58-4.61 *0.001***  **TT 60(30) 20(9.1)** |
| **rs10757278**  **AA 75(37.5) 165(75)**  **AG 97(48.5) 47(21.4) *0.001* A/G 0.62/0.38 0.86/0.14 62.82 3.70/2.65-5.18 *0.001***  **GG 28(14) 8(3.6)** |

OR: Odd Ratio, CI: Confidence Interval *The genotypic and allelic frequency distributions of polymorphisms between the groups were compared using x^2^ and HWE test. In all cases differences were considered significant at p< 0.05.


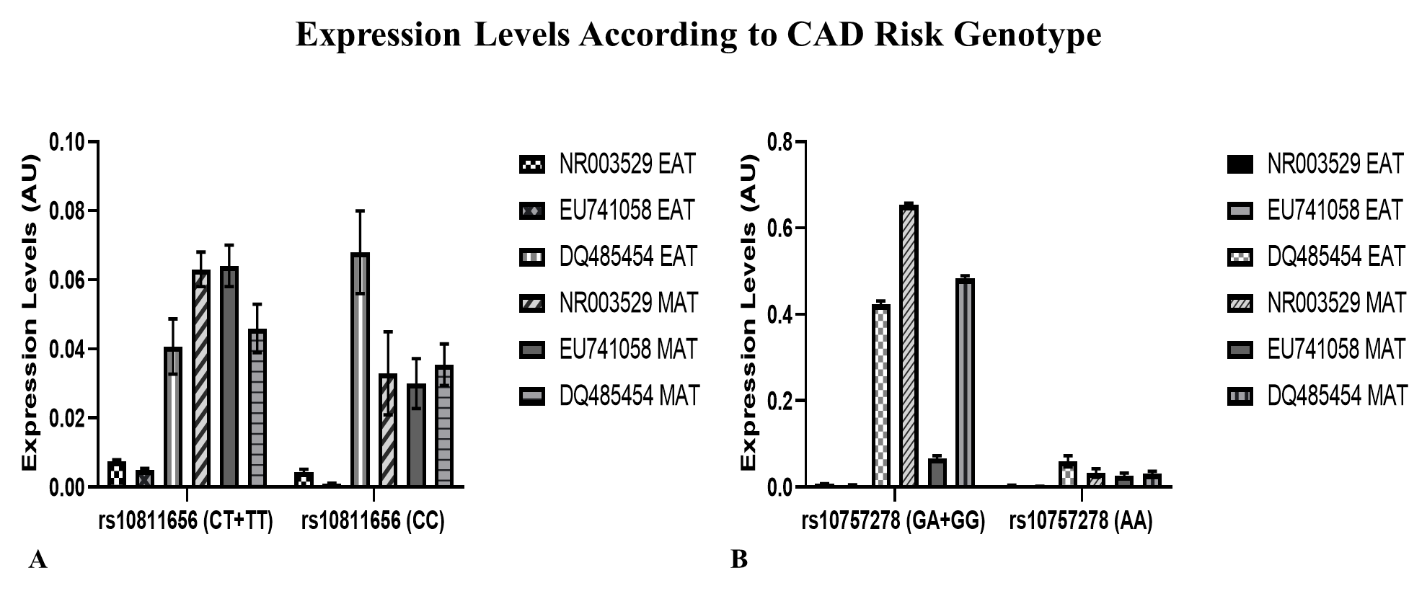


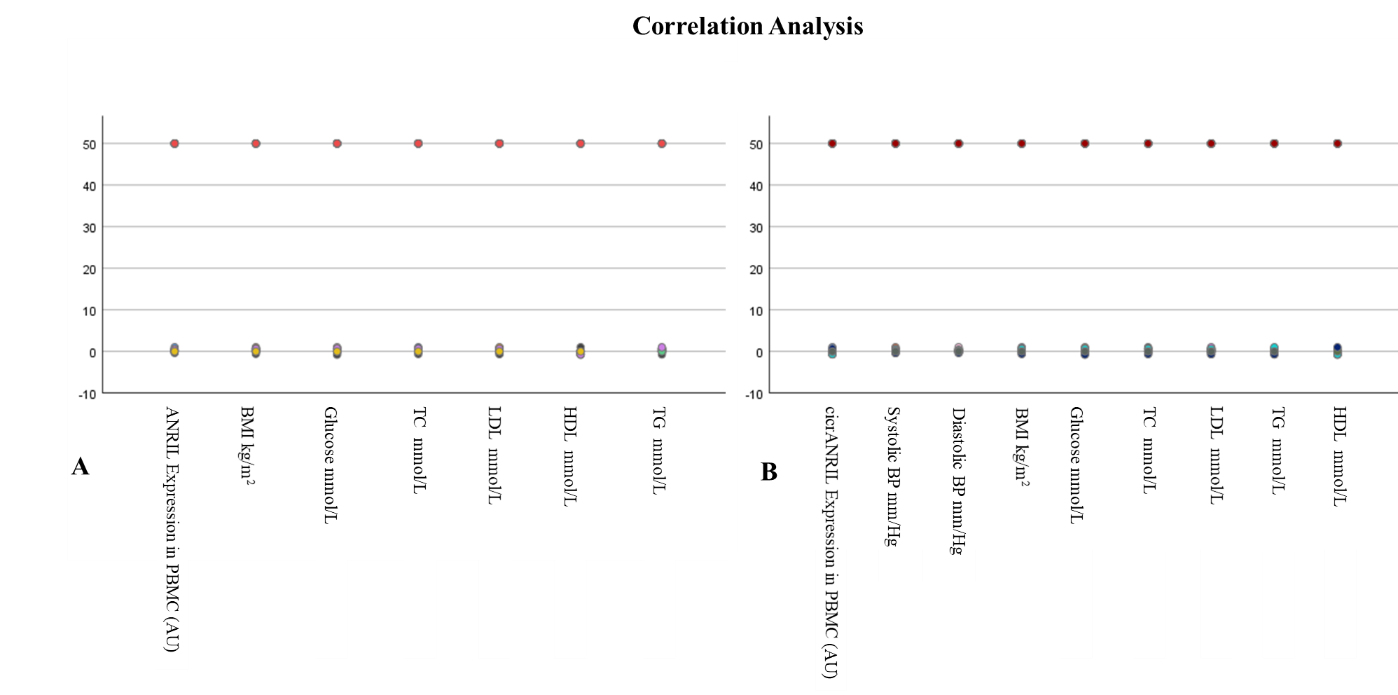
**Supplementary Figure 1:** The analyses of the expression levels of ANRIL linear transcript variants according to CAD risk genotypes. A: the results of rs10811656, B: the results of rs10757278. AU: Arbitrary Unit, EAT: Epicardial adipose tissue, MAT: Mediastinal adipose tissue

**Supplementary Figure 2:** The correlation analyses between expression levels of candidate genes and risk factors of CAD. A: the correlation analysis of ANRIL expression and CAD risk factors. B: the correlation analysis of circANRIL expression and CAD risk factors. PDMC: Peripheral Blood Mononuclear Cells, BMI: Body mass index, TC: Total cholesterol, LDL: Low density lipoprotein cholesterol, HDL: high-density lipoprotein cholesterol, TG: Triglyceride and BP: Blood pressure.
